# Transgender, Gender-Diverse, and Nonbinary Experiences in Physical Therapy: A Descriptive Qualitative Study

**DOI:** 10.1093/ptj/pzae086

**Published:** 2024-07-10

**Authors:** Madelaine Aird, Julie L Walters, Alex Ker, Megan H Ross

**Affiliations:** School of Allied Health & Human Performance, University of South Australia, Adelaide, South Australia, Australia; School of Allied Health & Human Performance, University of South Australia, Adelaide, South Australia, Australia; School of Social and Cultural Studies, Te Herenga Waka Victoria University of Wellington, Wellington, Wellington, New Zealand; RECOVER Injury Research Centre, The University of Queensland, Brisbane, Queensland, Australia

**Keywords:** Gender and Sexual Minorities, Gender Identity, Health, Physical Therapy, Transgender

## Abstract

**Objective:**

The objective was to explore experiences with and identify barriers and facilitators of utilizing physical therapy for people who identify as transgender, gender diverse, and nonbinary (TGNB).

**Methods:**

A qualitative descriptive design was employed using semistructured interviews conducted in New Zealand. Eligible participants were individuals who were 12 years old or older, who self-identified as TGNB, and who had accessed physical therapy at a community-based clinic that also provides a gender-affirming service. Participants were recruited via email invitation to the clinic database. Interview data were analyzed using reflexive thematic analysis. Demographics are reported descriptively.

**Results:**

Seventeen individuals (15–64 years old and identifying as 11 different genders) participated. All participants reported physical therapy experiences relating to 1 or more of the following 4 themes: challenging cisnormativity at policy, environmental, clinic, and therapist levels; safety and trust throughout the clinical experience, including clinic credibility for being a safe provider, clinic displays of TGNB inclusivity, implementation of safe clinic processes, and respectful therapist interactions; inclusive experiences in a clinic that provided affordable care and took active steps to understand and affirm TGNB identities and with physical therapists who had a high level of knowledge of TGNB-specific health issues and took a biopsychosocial approach to care; and sensitivity to body discomfort or dysphoria triggers. Barriers to and facilitators of care were identified at policy, environmental, clinic, and therapist levels.

**Conclusion:**

People who identify as TGNB face challenges to accessing safe and culturally sensitive physical therapy. However, there are achievable areas for improvement at policy, environmental, clinic, and physical therapist levels to gain trust and engagement in care for the TGNB community.

**Impact:**

This study provides a detailed exploration of TGNB physical therapy experiences and identifies specific areas of improvement for TGNB physical therapy care to provide clinicians and physical therapy clinics insights into the provision of safe and culturally sensitive physical therapy.

## Introduction

With an increased visibility of people who identify as transgender, gender diverse, and nonbinary (TGNB) in Australia and New Zealand,^1^^,^^2^ the need for TGNB-specific health care is rising.^3^ In Aotearoa (New Zealand), 2.5% of youths question their gender identity^4^ and 2.3% of Australians who are 15 to 17 years old identify as TGNB.^5^ Despite increased recognition of gender diversity in public and political discourse over recent years, including legal protections from gender-based discrimination,^6^^,^^7^ significant stigma and inequality remain.^8^ Health and access to health care are persistent areas of inequity. Individuals who identify as TGNB experience poorer physical and mental health than the general population,^9^^,^^10^ including a greater burden of disability, harassment, violence, suicide, suicidality, and homelessness.^11–13^

Globally, health inequities are recognized to emanate from social determinants of health,^14^ the economic and social factors that influence differences in health status. For individuals with TGNB identities this includes safe housing,^15^ income,^16^ schooling,^17^ and marginalization.^18^ Such determinants can be organized using the socioecological model of health,^19^ a framework for understanding the interconnections between interpersonal, organizational, environmental, and policy level factors that affect health. Strategies to improve health equity can be targeted to specific levels of the socioecological model of health to address and affect social determinants of health for the TGNB community.

Some, not all, individuals who identify as TGNB experience gender dysphoria: distress associated with the dissonance between a person’s gender and body.^20^ Exposing the body, observation, and physical touch are routine in the context of physical therapy,^21^ yet, despite a strong link between the physical body and physical therapy, there is a dearth of literature pertaining to TGNB experiences of physical therapy. Currently, there is no research exploring the factors that promote or inhibit the utilization of physical therapy care in this population. One paper investigating physical therapy experiences for the broader lesbian, gay, bisexual, transgender, queer, intersex, asexual and related communities (LGBTQIA+) found assumptions of gender, discomfort about touch and undressing, real and perceived discrimination, and lack of transgender specific health knowledge.^21^ However, only 10% of participants in the study identified as TGNB. Findings from other health professions suggest that people who identify as TGNB receive substandard levels of care.^22^ There are several explanations for this inequity in the literature: stigmatization, discrimination, restricted access to gender-affirming health care services, inadequate practitioner experience,^23^^,^^24^ and a lack of appropriate and culturally sensitive interactions.^25^ Therefore, an in-depth and specific investigation is required to understand the unique and nuanced experiences of physical therapy care for people who identify as TGNB.

Physical therapists in Australia indicate lacking sufficient knowledge levels to confidently and sensitively interact with people who identify as TGNB identities^26^ and physical therapy students report feeling unprepared and desire further training.^27^^,^^28^ Therefore, to provide insights to improve culturally responsive physical therapy for individuals who identify as TGNB, the 2 research questions for this descriptive qualitative study were as follows: What are the experiences of people who identify as TGNB with physical therapy in New Zealand? What are the barriers and facilitators to utilizing physical therapy for people who identify as TGNB in New Zealand?

## Methods

### Design

A qualitative descriptive design was chosen to provide easily comprehensible and readily utilizable insights into care experiences.^29^ Semistructured interviews were employed to realize the study’s aim of garnering detailed and nuanced descriptions of physical therapy experiences and the meanings attributed to them.^30^ A purpose-built semistructured interview guide (Suppl. Material 1) was developed collaboratively between the research team and Willis Street Physiotherapy (WSP) to allow exploration of topics while also allowing discovery of new concepts.^31^ Interview questions invited exploration of differences, if any, in care experiences between various clinics attended and were piloted with a member of the New Zealand transgender community.

### Theoretical Underpinnings

This study was underpinned by the theory of relativism (ie, all perspectives are individual and valid), such that people with TGNB identities were understood to have different experiences and, thus, there is no singular reality.^31^ This generated an approach that affirmed and explored diversity of experiences. The socioecological model of health was utilized for the categorization of barriers and potential solutions.

### Participant Selection

Eligible participants had accessed physical therapy services at WSP, were 12 years or older, self-identified or previously self-identified as TGNB, and were willing to participate in videoconferencing interviews. Participants were recruited via email sent to the clinic’s client database in December 2022, and study information was provided. Participants contact information and demographic data were collected via Qualtrics (Qualtrics; Provo, UT, USA; www.qualtrics.com). Informed consent of participants was obtained. Participant recruitment occurred until iterative analyses during data collection displayed adequate depth and repetition of concepts to substantiate the rigor of results.^32^

### Setting

Participants were clients who had attended WSP, a musculoskeletal clinic in Wellington, Aotearoa New Zealand, that provides a gender-affirming service (a fully subsidized and TGNB-specific service addressing musculoskeletal complaints arising from gender diversity, eg, wearing a binder). WSP values TGNB inclusivity and improvement of TGNB health outcomes, clinicians working in the gender-affirming service receive specific training in the area.

### Research Team

Analyses were conducted by M.A. (a physical therapy student who undertook qualitative training), M.H.R. and J.L.W. (qualified physical therapists who identify as LGBTQIA+ and are experienced qualitative researchers), and A.K. (a sociologist and Aotearoa New Zealand–based transgender health researcher).

### Data Collection

Data were collected between December 2022 and February 2023 using videoconferencing software (Zoom; Zoom Video Communications; San Jose, CA, USA; https://zoom.us/) and transcribed verbatim, with pseudonyms ascribed to preserve participant anonymity. An NZ$20 fuel voucher was provided to participants as an honorarium for their time. The funders played no role in the design, conduct, or reporting of this study.

### Data Analysis

Reflexive thematic analysis^33^ of qualitative data was guided by the 6 iterative stages described by Braun and Clarke.^34^ Themes and subthemes were generated inductively (ie, from within the data),^35^ and then once consolidated, subthemes were mapped to a socioecological model of health for organizational purpose. Throughout, the researchers made conscious efforts to reflexively question their own preconceptions and assumptions in interpretation.

First, M.A. read the full dataset and, secondly, generated preliminary codes. Next, the research team discussed potential themes to which M.A. categorized and grouped preliminary codes. Then, the research team discussed and refined themes and subthemes, and next, M.A. reread the entire dataset, coding any additional data. In the next stage, the researchers solidified themes and subthemes, and coding was finalized. Lastly, the research team defined the themes, and subthemes were then mapped to an adapted socioecological model of health. A researcher and member of the Aotearoa New Zealand transgender community (A.K.) further reviewed the analysis to ensure sensitivity to, and inclusivity of the Aotearoa New Zealand context. Any inconsistencies were discussed and either resolved or included in reporting, and data analyses were complete in March 2023. Demographic data were analyzed using descriptive statistics.

Study rigor was guided by Tracy’s 8 key markers for qualitative research quality.^36^ Strategies for rigor included ongoing researcher reflexivity, prolonged engagement from the research team, use of thick descriptions, peer examination and debriefing with A.K. Member checking occurred informally during data collection, (ie, the researcher conducting the interviews summarized and confirmed that their interpretation of participants responses was correct). Relevant criteria of the Consolidated Criteria for Reporting of Qualitative Research (COREQ)^37^ were satisfied.

### Role of the Funding Source

The funders played no role in the design, conduct, or reporting of this study.

## Results

### Participants

Of the 84 people responding to the screening questionnaire, 55 identified or previously identified as TGNB and 21 consented to be contacted for the interview. In total, 17 participants completed interviews. Participants were between 15 and 64 years old (median = 25) and identified with 11 different genders. More than three-quarters (76%) used they/them pronouns. The majority (88%) identified with a gender different from sex assigned at birth, none identified with an intersex experience (ie, born with a variation of sex characteristics). Most were of New Zealand-European ethnicity (88%), lived in metropolitan/urban areas (88%), and were employed full-time (47%) or students (24%). The sample varied regarding living situation and education level. Details of participant characteristics are provided in Table 1. Descriptions of gender identities are given in Supplementary Material 2.

**Table 1:** Characteristics of 17 Participants

**Characteristic**	**Value** ^*a*^
Age (y)	
Mean (SD)	27 (10.9)
Range	15–64
Gender identity*^b^*	
Nonbinary	11 (65)
Transgender	8 (47)
Transmasculine	5 (29)
Transfeminine	3 (17)
Genderqueer	3 (17)
Male	3 (17)
Agender	2 (12)
Female	2 (12)
Takatāpui (Māori Rainbow person)	1 (6)
Genderfluid	1 (6)
Prefer to self-describe: queer	1 (6)
Pronoun(s)	
They/them/theirs	9 (53)
He/him	3 (17)
He/him, they/them	2 (12)
They/them/ia*^c^*	1 (6)
She, they	1 (6)
She/her	1 (6)
Place of residence	
Metropolitan/urban	15 (88)
Regional	2 (12)
Ethnic group(s)*^b^*	
New Zealand European/Pākehā	15 (88)
Australian	1 (6)
Māori	1 (6)
European	1 (6)
Other	2 (12)
Living situation	
With housemates/friends	9 (53)
With partner	3 (17)
Alone	2 (13)
Other	3 (17)
Highest level of education	
University	6 (35)
Postgraduate	5 (30)
High school	5 (29)
Primary school	1 (6)

^a^ Values are reported as number (percentage) of participants unless otherwise indicated.

^b^ Participants were able to select multiple options.

^c^ In te reo Māori (the indigenous language of Aotearoa New Zealand), the pronoun for everyone is ia.

### Thematic Analysis

Four themes were identified during inductive analyses: challenging cisnormativity, safety and trust, inclusive experiences, and body discomfort or dysphoria (Tab. 2). Participants’ perspectives and beliefs about encounters (both positive and negative) and suggestions for improvements were spread across themes. Analysis indicates that participants experienced numerous interpersonal (ie, therapist-patient), organizational (ie, clinic), environmental (ie, context and cultural norms), and policy (ie, government recognition of gender) factors that influence physical therapy experiences and decisions to seek and/or return to care. Individual participant responses are differentiated by assigned pseudonyms.

**Table 2:** Theme Descriptions, Exemplar Quotes, and Socioecological Model of Health Level*^a^*

**Theme and Subtheme**	**Description**	**Exemplar Quote**	**Socioecological Model of Health Level**
Challenging cisnormativity	Reports of encountering assumptions of gender due to cisnormativity and binary gender norms.	“I get misgendered so often … you’re just coming out so often ... it’s a small emotional tax to be, ‘Actually my pronouns are he/him. Or I’m trans.’” (Siobhan, transmasculine)	Policy, environmental, and therapist: pervasive societal and cultural norms in relation to the gender binary.
Safety and trust	The level of psychological and emotional safety can establish or erode trust in care.		
Clinic credibility	Establishing clinic credibility for safe care can increase access.	“… trust is slow to build, as more people talk about it [a service] in their communities, you know it’s a safe, approved place to be.” (John, transgender, transmasculine, genderqueer, male)	Environmental: context of care, referral pathways, and inter-TGNB community assurance of safety.
Signposting and clinic processes	Signals of TGNB inclusivity can facilitate trust but must be accompanied by safe clinic processes.	“… seeing it [transgender pride flag] there, it’s like, ‘Oh, good. This feels like a place that’s for me.’” (Jessi, transgender, transfeminine, nonbinary) “… it doesn’t matter if the physio is culturally competent or good with this kind of stuff if the receptionist isn’t. It has to be the whole team.” (Sita, nonbinary)	Clinic: clinic displays of inclusivity that are followed by continuity of interclinic processes, such as pronouns and chosen name use.
Respectful therapists	Respectful interactions with physical therapists can facilitate feelings of safety and trust.	“… You want to be able to trust that [the physical therapist] won’t make weird comments about your body or the way you present.” (Jade, nonbinary)	Therapist: professional, nonjudgmental, and respectful therapist interactions.
Inclusive experiences	Experiences of inclusive, appropriate, and affordable care.		
Subsidized care	Clinics providing financial subsidies for care influence decisions to seek care.	“… whether I could continue [care] would be based on how much I could afford.” (Ellie, transmasculine, nonbinary)	Clinic: clinic services promoting equitable care.
Therapist knowledge of TGNB-specific health issues	Therapist knowledge, understanding, and training in TGNB-specific health issues.	“They [physical therapist] understood dysphoria. And I could say, ‘No, I’m feeling too dysphoric to do that,’ and they understood what that meant. I didn’t have to explain.” (Ashley, transmasculine, nonbinary)	Clinic and therapist: educational curriculum development, professional training, and individual therapist skills.
Biopsychosocial practice	Client-led biopsychosocial practice can result in feeling seen as a whole person and empowered in care.	“In the past, I’ve felt like I wasn’t treated as the expert in my own experience … [at WSP] every appointment feels like a collaboration.” (Rosie, nonbinary, agender)	Therapist: therapist abdication of control in care can enable client engagement and self-advocacy in care.
Body discomfort or dysphoria	Physical therapy may cause experiences of body discomfort or gender dysphoria.	“Anytime that I’m going to be talking about my body … especially parts of my body that I really dislike, it’s always going to be hard.” (Ellie, transmasculine, nonbinary)	Therapist: therapist sensitivity to triggering factors can minimize the impact of body discomfort or dysphoria.

^a^ TGNB = transgender, gender diverse, and nonbinary; WSP = Willis Street Physiotherapy, a musculoskeletal clinic in Wellington, New Zealand, that provides gender-affirming service.

#### Theme 1: Challenging Cisnormativity

The theme challenging cisnormativity encompassed reports of erroneous assumptions or erasure of participants gender identity due to societal norms of cisnormativity and the gender binary. Participants discussed encountering only binary options (ie, male/female) for sex and/or gender on governmental institution paperwork (eg, compensation claims) and clinic intake forms. Others experienced perceptions of binary gender in previous physical therapy encounters. For example, 1 participant said a physical therapist (not at WSP) talked about their body “in terms of male and female [with regard to] exercises, muscle tone, strength-based things … I definitely got misgendered … it was definitely a very gendered view.” Responses to these experiences were largely consistent. Participants described feeling exhausted (by repeated misgendering), “invisible,” and “other[ed].” Many expressed frustration that the onus was on them to provide education (eg, on pronouns).

At WSP, assumptions were largely not encountered, and this was attributed to staff using gender-neutral greetings, nongendered language (when appropriate), self-describe/multiple gender options, and space for pronouns/chosen name on intake forms. Generally, participants described feeling surprised (at the rarity of the experience), validated, and uplifted (because they were recognized) when this happened. Several participants discussed wishing this was the case within physical therapy broadly and suggested therapists should receive cultural sensitivity training that encompasses reflexivity of gender perceptions to address this—“… often it’s people’s unexamined views (of gender) that are harmful. Or they hold a view, but they might not know why.”

#### Theme 2: Safety and Trust

The most common theme, safety and trust in care, encompassed reports of trusting services and providers to deliver respectful, and psychologically and emotionally safe care. Subthemes were clinic credibility, signposting and clinic processes, and respectful therapists.

Clinic credibility, in relation to safety, was described by many participants as crucial in informing clinic choice. Referrals from trusted services (eg, gender-affirming medical services), affiliations with TGNB-friendly community organizations, and inter-TGNB community endorsement was reported as contributing to WSP’s credibility for being a safe clinic—for example, “… I’m always going to ask a queer friend what their experience is before trusting [a service].” Such endorsements helped participants overcome distrust from previous negative health care experiences, as 1 participant said, “… it [is] hard to shelve my previous experiences (of transphobia); there’s always [a] level of paranoia and anxiety.” Some participants also expressed feeling safer (to access care) at WSP because it resided within a “safe little Wellington queer bubble” of TGNB acceptance (the Wellington region has the highest proportion of LGBTQIA+ people in New Zealand relative to its population and is well known within the community as being “queer-friendly”). Participants anticipated this would not be the case in rural or remote settings.

The second subtheme encompassed signposting and clinic processes that displayed and ensured TGNB inclusivity at WSP. Signposting included displaying transgender and nonbinary pride flags, inclusivity statements and website links to other gender-affirming health services. Signposting facilitated trust in WSP and promoted care-seeking behavior—for example, “… the fact that there’s little indicators of how you’ll be treated before you have to take that step [to seek care] makes it a lot easier to do.” Notably, a substantial number of participants expressed concerns for “virtue signalling” and emphasized the importance of safe clinic processes accompanying signposts. Examples of safe clinic processes included signage “to use the bathroom that best aligns with your gender”, identifying locations of nearest gender-neutral bathrooms, and continuity of pronoun and correct name use by all staff members and within interorganizational documentation and data management (preventing “deadnaming”). One participant described, referencing a past health care encounter: “… it almost makes it worse when you have people that are really affirming … and then you have someone within the same service that isn’t … it makes it a lot more jarring.”

The third subtheme involved reports of interactions with respectful therapists at WSP that enabled participants to feel safe to be their whole selves within consultations and disclose their TGNB identity (if relevant). In previous physical therapy encounters, many participants described initial apprehension about being judged by physical therapists because of their TGNB identity—for example, wondering, “… are they [the physical therapist] going to be accepting? Are they okay with it?” Several said having to conceal their TGNB identity had a negative impact on their engagement with care—“if I can’t be fully open and comfortable about who I am … I’m not going to get the most out of what I need.” Some (particularly younger) participants said they felt uncomfortable disclosing without therapists establishing adequate rapport.

Mostly participants described feeling safe to disclose/discuss their TGNB identity with physical therapists at WSP, who were repeatedly described as professional and friendly. Examples of respectful therapist interactions included stating their own pronouns when introducing themselves, using participants correct pronouns, and addressing gender identity and expression with nuance, which appeared to normalize gender diversity—1 participant recounted, physical therapists “not acting like being trans is weird is always validating … just brining up (in consultations) binding or other aspects of being trans very matter-of-factly, without a change in tone.”

#### Theme 3: Inclusive Experiences

The theme inclusive experiences comprised descriptions of inclusive, appropriate, and affordable physical therapist experiences at WSP. The subthemes were subsidized care, therapist knowledge of TGNB-specific health issues, and biopsychosocial practice.

Subsidized care (for individuals who identify as TGNB) featured strongly in participant responses and provoked positive and appreciative reactions. Cost of care was described as “… so prohibitive” and was a decisive factor in most participants decisions to seek and/or continue care. A small number emphasized this was particularly important given the high “cost” of being TGNB (eg, gender-affirming surgery costs, purchasing new clothes, mental health expenses etc.).

At WSP, participants noted and valued the high standard of therapist knowledge of TGNB-specific health issues. This included understanding binding, gender dysphoria, fearing not “passing”, the importance of gender expression, and basic TGNB terminology. However, this was not always the case. Some participants described experiencing frustration, primarily because they did not feel the WSP physical therapists’ questions about hormone medications were relevant—for example, wondering, “do they (physical therapists) need to know if I’m on testosterone?” One participant described an historical encounter, not at WSP, where a physical therapist dismissed their symptoms—saying their physical therapist “… thought, ‘Oh, you’re trans, so how can you have pelvic floor issues?’” Many participants said that physical therapists broadly do not have the required level of knowledge to work appropriately with the TGNB community—for example, in the past, “I’ve had to do quite a bit of educating (of physical therapists) about the effects of hormones on the body.” Most participants expressed a desire for increased therapist training on TGNB-specific health issues, especially gender-affirming hormone therapy, as it related to muscle bulk.

The last subtheme, biopsychosocial practice delivered by WSP physical therapists, was especially prominent in most participants responses. Consultations at WSP were consistently described as “client led,” and participants felt they were “given a lot of control in [their] own experience.” Participants expressed feeling that their whole selves were seen and “really understood” because multiple aspects of their lives (eg, mental wellbeing, personal preference, individual circumstances) were considered in treatment and management. For example, 1 participant said this approach felt “… integrated into a whole human life.” Participants reported this engendered their trust in WSP physical therapists, making therapeutic intervention both engaging and more easily implementable into their lives.

#### Theme 4: Body Discomfort or Dysphoria

Finally, several participants discussed body discomfort or dysphoria due to touch, exposure, and observation of the body in physical therapy consultations. For example, saying, “… the [physical therapist] watching me closely, [made] me very aware that my body doesn’t look as I’d like it to look and that someone else is also seeing it in a way that doesn’t feel correct to me.” WSP therapist factors that increased participant comfort were confirming anatomical terminology that clients use—“… using chest over any feminine words”—and sensitivity to shared gym spaces and mirrors during consultations—“coming across an unexpected mirror can trigger some dysphoria”—particularly early in gender affirmation. Many participants reported physical therapists gaining continual and clear consent for touch was expressly important. For example, the WSP physical therapist “always asked for consent” and this was “really helpful.” Other positive factors included sensitivity to draping, consult room privacy, and warning prior to disrobing so participants could mentally and physically prepare (ie, via clothing choice). Some also said focusing on therapy outcomes (opposed to the physical body) increased their comfort. A small but notable number of participants discussed past sexual trauma associated with specific body parts/areas and suggested that therapist understanding of and sensitivity to this was valuable.

## Discussion

Our study is the first to investigate experiences of physical therapy and the factors affecting its utilization for people who identify as TGNB. Although physical therapy encounters presented challenges to inclusivity for individuals who identify as TGNB, there were employable strategies to overcome these (Figure). The key finding of our study is that the most important factor in having a positive physical therapy experience and in facilitating the utilization of physical therapy is the feeling of safety and inclusion throughout the clinical encounter. Most participants had largely positive encounters within a clinic where specific steps toward TGNB inclusivity were taken. These steps broadly fit into the themes: challenging cisnormativity, safety and trust, inclusive experiences, and body discomfort or dysphoria. Some negative experiences were also reported, predominantly related to fears around psychological safety of attending physical therapy, rather than WSP itself. The following discussion summarizes each theme and offers specific recommendations for areas of improvement in physical therapy care delivery at each level of the socioecological model of health, as illustrated in the Figure.

**Figure:**
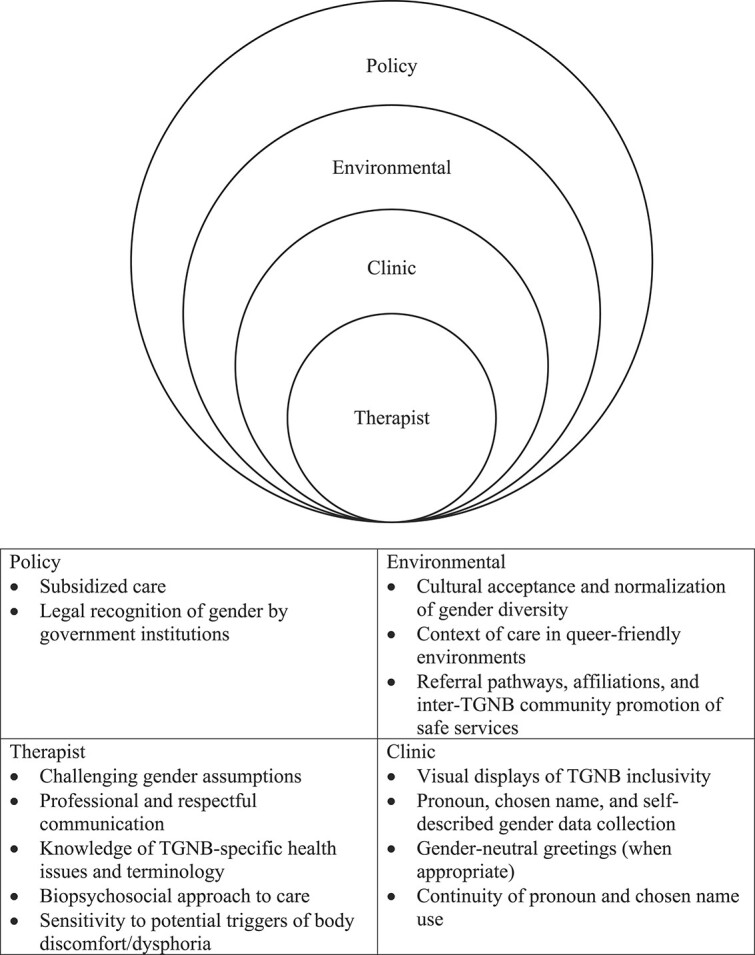
Areas for improvement in physical therapist care delivery at each level of the socioecological model of health. TGNB = transgender, gender diverse, and nonbinary.

Our findings were consistent with previous research focusing on the broader LGBTQIA+ community, in that there is pervasive cisnormativity in physical therapy.^21^^,^^26^ As with other health care settings,^38–40^ we found misgendering and marginalization (which may attenuate minority stress^41^ and negatively affect TGNB health outcomes^42^) can occur in physical therapy but was noticeably absent at WSP, where our participants attended. Our study found gender recognition at the clinic level was crucial to inclusion. Like other findings, participants in our study supported inclusive clinic intake forms (eg, chosen name, self-describe gender options) and pronoun data collection.^43^ Although many physical therapists in Australia hold largely egalitarian attitudes toward LGBTQIA+ patients,^44^ our study found physical therapists can inadvertently misgender patients. Therefore, educational training that challenges therapist cisnormativity is 1 mechanism for changing physical therapy culture to be more inclusive of gender diversity.

Feelings of psychological safety depended upon building trust across all touchpoints of the clinical journey. Most participants expressed a fear or anxiety in seeking care because of poor past health care experiences (predominantly within other settings). As real or perceived discrimination is consistently reported as a barrier to TGNB access and underutilization of health care^24^^,^^45–48^ our study’s finding that safety needs to be established at environmental (ie, contextual, and social credibility of a clinic for safe care), clinic and therapist levels would facilitate care-seeking and continued engagement in care.

Having inclusive experiences may help gain the TGNB communities’ trust of health care. In our study, therapists’ biopsychosocial approach to practice and increased knowledge of TGNB-specific health issues appeared to gain participants trust. Our participants supported the utilization of client-centered care, which is largely considered the gold standard,^49^ and can promote both client self-management^50^ and empowerment.^51^ We found high levels of physical therapy training in TGNB-specific health issues within WSP, but not in previous physical therapy experiences, suggesting the need for increased therapist training. Current research suggests health care provider training in TGNB care can improve health outcomes and increases competence in working with the TGNB community.^52^ In consideration of the well-documented stigmatization and systemic discrimination against people who identify as TGNB,^53^ the onus is on the physical therapy profession to instigate changes in care, specifically the biopsychosocial aspects of care. Lack of trust in health care encounters is shown to compromise effectiveness of therapy outcomes and can lead to later health care avoidance.^54^^,^^55^ Conversely, respectful and trusting therapeutic relationships can improve mental wellbeing for individuals who identify as TGNB.^56–58^ As lack of trust in health care is a persistent barrier for this population,^59^ physical therapists who tailor care to the individual and demonstrate understanding of individual clients’ contexts, socioemotional and health needs are likely to facilitate culturally responsive care. Furthermore, our findings highlight that affordability was positively associated with physical therapy access. The myriad structural and financial barriers to accessing health care faced by the TGNB community are well established^23^ and include socioeconomic disadvantage^12^ and employment discrimination^60^ (the unemployment rate of TGNB Australians is 4-fold that of the general population,^1^ and in Aotearoa New Zealand, the disposable income of people who identify as TGNB is one-third lower than that of people who identify as cisgender).^16^ As cost was the final barrier to access for our study’s participants, it appears fee subsidization is a key factor in increasing physical therapy utilization. For equitable health care provision, policy level changes to health care costs are required, however, where possible, clinic level measures (eg, subsidized sessions at WSP) can demonstrate allyship and commitment to equity.

Finally, participants in our study supported professional education for basic TGNB terminology and awareness of specific areas of the body and/or gender representation that may present psychological triggers for the community. Our findings of the potential for physical therapy to trigger body discomfort and dysphoria provides insight into specific areas of consideration when working with people who identify as TGNB. Physical therapist sensitivity to factors that could provoke discomfort/dysphoria and adoption of trauma-informed approaches to care (shown to be beneficial in other health care contexts^61^) increased participant comfort when this was important. Although there is a lack of physical therapy-specific TGNB literature pertaining to physical examination, some nonacademic publications (primarily in primary care) exist.^62^ Profession-specific training to increase knowledge of such factors at undergraduate and professional levels offers a way forward to improving care for individuals who identify as TGNB.

### Limitations

The transferability of our findings requires several considerations. Our study was conducted in Aotearoa New Zealand which, like Australia, has relatively progressive legislative and social acceptance of people who identify as TGNB. Findings are likely most applicable to similar socioeconomic and political contexts. Our study incorporated a diversity of participants (varied in age and education levels), and findings are likely to be representative of the Aotearoa New Zealand context. However, sampling within WSP may underrepresent TGNB experiences and perspectives of general physical therapy—participants in this study, by majority, only sought physical therapy care at WSP. Despite this, we were able to obtain information about strategies that fostered inclusion and positive physical therapy engagement. Further research into the development and effectiveness of profession-specific TGNB health educational resources would benefit the physical therapy profession.

Our findings highlight that people who identify as TGNB could experience inclusive and respectful physical therapy when client-centered care was delivered by clinics and clinicians who were affirming of TGNB identities. Access to safe, respectful, and affordable physical therapy would play a role increasing the health status for this population and promote future care engagement. Physical therapists have a moral and professional obligation to provide care that is tailored to the individual and to create welcoming and inclusive care for all. An improved awareness and education of TGNB-specific barriers to health care and health issues offer ways forward for improving TGNB physical therapy experiences.

## Supplementary Material

2023-0615_R2_Supplementary_Material_1_pzae086

2023-0615_R2_Supplementary_Material_2_pzae086

## Data Availability

Data are not available due to ethical restrictions.
