# Supplementary material for: Transgender, Gender-Diverse, and Nonbinary Experiences in Physical Therapy: A Descriptive Qualitative Study
Source: Phys Ther. 2024 Jul 10;104(10):pzae086. doi: 10.1093/ptj/pzae086 (PMC11524892; doi:10.1093/ptj/pzae086)
Supplement: 2023-0615_R2_Supplementary_Material_1_pzae086 [file 2023-0615_r2_supplementary_material_1_pzae086.pdf]

Supplementary Material 1. Semi-structured interview guide.

1. Can you please describe your history with physical therapy to date?
    a. Probes/Prompts
        i. *How many different clinics, how many different practitioners, over what period of time (in relation to transition / gender identity)*
        ii. *What was your main reason for attending?*
2. Did you have any expectations of physical therapy before you went? If so, what were they?
    a. Probes/prompts
        i. *Where did they come from? Why did you think/expect that?*
        ii. *Did it impact your decision about going? (when, where, who)? How?*
        iii. *Did your experience match your expectations? If so, why/how? Why not?*
3. Can you tell me about any negative experiences you had with physical therapy?
    a. Probes/Prompts
        i. *Ask about making appointment, entering clinic, clinic space, staff, consultation, facilities*
        ii. *What made this a negative experience?*
        iii. *How did it make you feel?*
        iv. *Did you go back? Why/why not?*
4. Can you tell me about any positive experiences you had with physical therapy?
    a. Probes/prompts
        i. *Ask about making appointment, entering clinic, clinic space, staff, consultation, facilities*
        ii. *What made this a positive experience?*
        iii. *How did it make you feel?*
        iv. *Did you go back? Why/why not?*
5. Can you describe your experience at Willis Street Physical therapy?
    a. Probes/prompts
        i. *Why/how did you choose this practice?*
        ii. *Was it different to other physical therapy clinics? If so, how?*
        iii. *Were you aware this clinic offers a sex or gender-affirming service? How did you find out? Did that influence your decision, how?*
        iv. *Did you feel safe? Why/how or why/how not?*
        v. *Did you feel the physical therapist had the expertise / knowledge needed for you? Why/why not? Did you like the physical therapist? Why/why not?*
        vi. *Could they have done anything differently? (the clinician and clinic)*
        vii. *Would you refer someone? Why, why not?*
6. What do you wish your physical therapist (or health professionals) knew about the sex and/or gender diverse community?
    a. Probes/prompts
        i. What do you think is important for physical therapists to know?
        ii. Was there anything you have had to teach physical therapists? If so, what?
7. Is there anything else you would like to add?
