# Supplementary material for: Transgender, Gender-Diverse, and Nonbinary Experiences in Physical Therapy: A Descriptive Qualitative Study
Source: Phys Ther. 2024 Jul 10;104(10):pzae086. doi: 10.1093/ptj/pzae086 (PMC11524892; doi:10.1093/ptj/pzae086)
Supplement: 2023-0615_R2_Supplementary_Material_2_pzae086 [file 2023-0615_r2_supplementary_material_2_pzae086.pdf]

Supplementary Material 2. Glossary of gender terminology. Adapted from the Australian Human Rights commission,[63] Mental Health Foundation of New Zealand[64] and Australian Institute of Family Studies.[65]

Term	Definition
Agender	A term describing people who identify as being without gender or having a gender that is neutral.
Binding	Varying methods used to flatten the chest area, usually with the goal of reducing feminine appearance.
Cisgender/cis	A term describing people whose gender identity aligns to sex assigned (or assumed) at birth.
Cisnormativity	A discourse or ideology based upon the assumption that all people identify as the gender assigned at birth, privileges this over other forms of gender identity, rendering people who do not as 'abnormal' or incorrect.
Deadnaming	The act of referring to a transgender or nonbinary person by a name they used prior to transitioning. i.e. their birth name.
Gender Affirmation	An umbrella term for a non-linear process of socially, medically, or legally living and thriving in the gender a person identifies with.
Gender Binary	A socially constructed view in which gender falls into two rigid categories of male and female.
Gender Diverse	An umbrella term describing anyone who does not identify as cisgender, representing a diversity of gender identities beyond binary gender.
Gender Dysphoria	Refers to discomfort or distress associated with the discrepancy between a person's gender identity and the persons assumed sex at birth.
Gender Expression	Refers to a person's presentation, including physical appearance and behavioural that communicates aspects of gender or gender role. It may or may not conform to a person's gender identity.
Gender Identity	An inner sense of one's gender which may be within, moving around between, a blend of or outside of the gender binary. It is internal and not necessarily visible to others.
Genderfluid	A person who does not identify with a single fixed gender or has a moving or flexible gender identity.
Genderqueer	A person whose gender identity lies outside of the gender binary (ie. identifies with none or both genders).
Microaggression	Inadvertent, unintentional, often indirect instance of discrimination due to pervasive assumptions of cis-normativity and assumptions of binary gender.
Misgendering	An interaction in which a person is intentionally or unintentionally referred to with the incorrect pronouns.
Nonbinary	Refers to individuals whose gender identity is outside of male/female gender norms. An individual may identify solely as nonbinary or relate to nonbinary as an umbrella term and consider themselves genderfluid, genderqueer, transmasculine, transfeminine, agender, bigender, or something else.
Passing	Being correctly perceived in social encounters as the gender that a person identifies with.
Takatāpui	A Māori language term that is used similarly to LGBTIQA+ and denotes people who identify with diverse genders and sexualities.
Trans or transgender	Refers to individuals whose gender identity is different from sex assigned at birth. Trans people may position 'being trans' as a history, or experience, rather than an identity, and consider their gender identity as simply female, male, or nonbinary.
Transmasculine and transfeminine	A term describing a transgender person assumed to be a boy/girl at birth but identifies or expresses themselves towards the masculine/feminine end of the gender spectrum.
Queer	An umbrella term to describe a range of sexualities and gender identities outside of heterosexual and binary gender norms.

References

63. Glossary of Terms. Australian Human Rights Commission; 2018. https://www.hrc.org/resources/glossary-of-terms

64. Kerekere E. *Takatāpui: Part of the Whānau*. 3 ed. Tiwhanawhana Trust and Mental Health Foundation; 2015.

65. LGBTIQA+ glossary of common terms. The Australian Institute of Family Studies; 2022. https://aifs.gov.au/resources/resource-sheets/lgbtiqa-glossary-common-terms
